# Supplementary material for: Values and preferences related to workplace mental health programs and interventions: An international survey
Source: PLoS One. 2023 Sep 27;18(9):e0283057. doi: 10.1371/journal.pone.0283057 (PMC10530006; doi:10.1371/journal.pone.0283057)
Supplement: S1 File — (PDF) [file pone.0283057.s001.pdf]

Online survey to support the development of the WHO mental health and work guidelines: values and preferences of programs and services for the promotion, prevention and support of mental health at work

Survey Flow

Consent Form: **Online survey to support the development of the WHO mental health and work guidelines: values and preferences of programs and services for the promotion, prevention and support of mental health at work**

Principal Investigators:
Andrew J. Greenshaw
Department of Psychiatry, University of Alberta
Email: andy.greenshaw@ualberta.ca

Jill Murphy, Ph.D.
Department of Psychiatry, University of British Columbia
Email: jill.murphy@ubc.ca

Welcome to the online survey to support the development of the WHO mental health and work guidelines: values and preferences of programs and services for the promotion, prevention and support of mental health at work

Introduction: You are invited to participate in a short, anonymous survey for working people 19 years and older that is part of a research study on workplace mental health interventions (services, activities, or programs). With your participation in the survey, the research team hopes to better understand what your values and preferences are (what you feel is important and how strongly you feel about it) about workplace mental health issues, services and programs. **The results of this survey will be used to inform a World Health Organization (WHO) guideline for mental health and work.**

Who is conducting the study? This work is being done in collaboration between the University of British Columbia, University of Alberta

Inclusion criteria: Paid workers over the age of 18 years old.
Exclusion criteria: Unpaid workers, military personnel, and any individuals under the age of 18 years.

Study Procedures: If you agree to take part in this study, you will be asked to complete a survey (20-30mins) about workplace mental health interventions.

Benefits and Risks: You will receive no direct benefits from participating in this research study. Your responses may help us better understand your values and preferences around workplace mental health issues, services, and programs.

There are no foreseeable risks involved in participating in this study beyond those encountered in day-to-day life. However, some of the questions in this survey may remind you of something that happened at work that made you feel negative emotions (for example, sadness, anger, stress). If this happens, you may choose to stop the survey at any time.

If you are experiencing severe distress, please seek help from your local emergency services

or health provider.

Confidentiality and Data Security: This survey is administered on Qualtrics. This platform has safeguards to ensure that your responses stay confidential and restricted to authorized personnel. Qualtrics does not collect personal information such as name, email address or phone number. The survey does not ask you for any information that can identify you. Therefore, your responses will remain anonymous. No one will be able to identify you or your answers.

Responses from all online and paper surveys will be entered into a statistical software for analysis, and will be kept on a password-protected, secure server. All responses will be deleted 10 years after data analysis has been completed. The data gathered may be looked at again to help us answer other study questions. If so, an ethics board will ensure that the data are used ethically.

Remuneration: There is no remuneration for participating in this survey.

Voluntary Participation/Ability to Withdraw: Your participation in this survey is voluntary. You may refuse to take part in this study, or exit the survey at any time, without penalty. You are free to skip answering any questions you do not wish to answer for any reason. If you choose to stop the survey early by clicking the “STOP SURVEY” button provided at the bottom of the screen, the information you provided will be considered withdrawal of consent and will not be used by our study team. Please note: Once you complete the survey and submit it, you will not be able to withdraw your consent as the survey is anonymous and the research team would not be able to identify which submitted survey was yours.

Study Questions: If you have questions about the study or are interested in the findings, you may contact Dr. Jill Murphy at jill.murphy@ubc.ca.

Conflict of Interest: The research team have no known, actual, apparent, potential, or perceived conflicts of interest in conducting this study.

Ethics Questions or Concerns about the Study: If you have any questions about your rights as a participant, or any concerns about your experiences while participating in this study, contact the University of Alberta Research Ethics Office at (+1) 780-492-2615.

By completing the survey, you are consenting to participate in this research.

- I have read and understood the subject information and consent form of this study.
- I understand that my responses will be anonymous, and that the information collected will be only used for scientific objectives.
- I understand that my participation in this study is voluntary and that I am completely free to refuse to participate or withdraw from this study at any time.
- I understand that I am not waiving any of my legal rights as a result of clicking yes below to continue to the survey.
- I understand that there is no guarantee that this study will provide any benefits to me.
- I have read this form and I freely consent to participate in this study.

After reading the information above, do you agree to participate in this survey?

▼ Yes ... No

Definitions:

Definitions:

Below are definitions used in this survey. You can access the definitions to these terms again throughout this survey by placing your mouse/cursor over any terms with a star ("*") next to them.

Mental health* A state of well-being in which every individual realizes his or her own potential, can cope with the normal stresses of life, can work productively and fruitfully, and is able to make a contribution to their community.

Mental health services or programs* Activities or interventions that seek to promote or support mental health (e.g. wellness seminars, stress management, mindfulness, employee assistance programs, return to work, etc.)

Mental health challenges or issues* Mental health issues or challenges may include diagnosed mental health disorders (e.g. depression, anxiety, bipolar disorder, etc.) or refer to challenges that are not diagnosed but still impact a person's mental wellbeing (e.g. too much stress, burnout, etc.). Both diagnosed and undiagnosed mental health challenges might impact a person's work - for example, it might be harder to focus, meet deadlines, or mean they take more sick days.

Employee Assistance Programs* Employee assistance programs are programs that may be offered to workers that aim to help people balance work and life issues. They typically offer support and counseling to help people deal with stress, family issues and more.

Stigma* A mark of disgrace that sets a person apart from others.

Q1.1 **Section 1 of 4: This section asks you for information about you (your age, gender, education, etc.)**

Q1.1 What is your age? (example: 36)

____

Q1.2 What is your gender?

- ○ Woman
- ○ Man
- ○ Transgender
- ○ Other, please specify ____
- ○ Prefer not to say

Q1.3 What country do you currently live in?

▼ Afghanistan ... Zimbabwe

Q1.4 What is the size of the community that you live in?

- ○ Large city (>1 million inhabitants)
- ○ Medium city (300,000-1 million inhabitants)
- ○ Small city (100,000 - 300,000 inhabitants)
- ○ Large town (20,000-100,000 inhabitants)
- ○ Medium town (1,000-20,000 inhabitants)
- ○ Small town, village, or hamlet (
- ○ Other, please specify ____

Q1.5 What is the highest level of formal education you have completed (check one box):

- ○ None
- ○ Primary school
- ○ Secondary/high school (including vocational high school)
- ○ University Undergraduate Degree (e.g., Bachelor Degree)
- ○ Graduate Degree (e.g., Master or Doctor Degree)
- ○ Other, please specify ____

Q1.6 Do you identify as being a member of a sexual or gender minority group in the country where you live/work?

- ○ Yes
- ○ No
- ○ Prefer not to say
- ○ Other, please specify ____

Display This Question:

If Q1.6 = Yes

Q1.6a As a member of a sexual or gender minority group, how do you identify? (If you would prefer not to say, please leave blank)

____

Q1.7 Do you identify as being a member of a racial or ethnic minority group in the country where you live/work?

- ○ Yes
- ○ No
- ○ Prefer not to say
- ○ Other, please specify ____

Display This Question:

If Q1.7 = Yes

Q1.7a Do you identify as being a member of an Indigenous group in the country where you live/work?

- ◯ Yes
- ◯ No
- ◯ Prefer not to say
- ◯ Unsure
- ◯ No

Display This Question:

If Q1.7 = Yes

Q1.7b As member of a racial or ethnic minority group, how do you identify? (If you would prefer not to say, please leave blank)

____

Q1.8 Do you have caregiving responsibilities at home? (e.g., for children, parents, and/or other adults)?

- ◯ Yes
- ◯ No

Q1.9 What is your current employment status?
(check all that apply)

- ☐ Full-time paid employment
- ☐ Part-time paid employment
- ☐ Self employed/ small business owner
- ☐ Family business
- ☐ Casual, seasonal, or temporary
- ☐ Informal
- ☐ Crowdsource
- ☐ Gig/platform/freelance/contract work (includes zero-hour contracts)
- ☐ Sickness-leave/disability leave
- ☐ Other, please specify

____

Q1.10 Where do you primarily do your work duties?

- ○ From home
- ○ At a work-site location
- ○ Other, please specify ____

Q1.11 Do you work for the public or private sector?

- ◯ Public sector
- ◯ Private sector (for-profit)
- ◯ Private sector (not-for-profit)
- ◯ Other, please specify ____

Q1.12 How many people work for your employer or business?

- ◯ 1-9
- ◯ 10-19
- ◯ 20-49
- ◯ 50-249
- ◯ 250+
- ◯ Self-employed/owner, no staff (you are the only employee)
- ◯ I don't know

Q1.13 What best describes the type of work you do?
Industry
More detail

▼ Type ... Other, please specify. ~ Other, please specify.

Display This Question:

If Q1.13 = Other, please specify.

Q1.13a If you chose "other," please specify what type of work you do here:

____

Q1.14 Are you responsible for promoting and/or supporting the mental health of workers, including via risk assessment and management of psychosocial risks in the work environment?

○ Yes

○ No

Display This Question:

If Q1.14 = Yes

Q1.14a Do you work in any of the following roles related to promoting, preventing, and/or supporting mental health for people who work?
(check all that apply)

▢ Mental health worker

▢ Occupational health/health and safety worker

▢ Human resources

▢ Employee assistance program provider

▢ Union representative

▢ Other, please specify

____

Q1.15 Does your work match any of the following services?
(check all that apply)

- ☐ Health worker (e.g., physician, nurse, etc.)
- ☐ Emergency public services work (e.g., firefighting, paramedic, police)
- ☐ Humanitarian work (national staff)
- ☐ Humanitarian work (international staff)
- ☐ No/Not applicable

Q1.16 Which of the following best describes your position?

- ○ Someone supervises me, I supervise no one
- ○ Someone supervises me, I supervise one or more people
- ○ No one supervises me, I supervise no one
- ○ No one supervises me, I supervise one or more people

Q1.17 Do you identify as living with a disability?

- ○ Yes
- ○ No

Q1.18 Do you and/or have you ever experienced a mental health challenge or issue?

- ○ Yes
- ○ No

Q1.19 Are you currently and/or have you ever taken a formal absence or leave from work for a mental health issue or challenge, including due to stress?

- ◯ Yes
- ◯ No
- ◯ Prefer not to answer

Q1.20 Are you currently and/or have you ever been supported to return to work after a mental health related issue or challenge, including due to stress (e.g., through a formal Return to Work program that supported you to go back to work)?

A Return to Work program is a plan that helps workers safely return to suitable work, usually after a period of sickness absence.

- ◯ Yes
- ◯ No
- ◯ Unsure
- ◯ Other, please specify ____
- ◯ Prefer not to answer

Q1.21 Have you ever been supported to find work because of a mental health related issue or challenge (e.g., through a formal program that helped you to find new work)?

- ◯ Yes
- ◯ No
- ◯ Unsure

Q1.22 Does your work offer benefits (e.g., health insurance, vacation time, paid sick days, social protection, etc.)?

- ○ Yes
- ○ No
- ○ Unsure

Q1.23 Does your work have links or connections with mental health programs and services that you can access outside of work and/or working hours (resources supported by your work that you can access outside of work or after hours if you needed it)?

- ○ Yes
- ○ No
- ○ Unsure

End of Block: Section 1: Demographics

Start of Block: Section 2: Values and Preferences - General Questions

2.1 **Section 2 of 4: This section asks questions about what you think is important about mental health, and mental health programs and services at work generally.**

What type of impact does your work have generally on your mental health?

- ○ Very positive
- ○ Somewhat positive
- ○ Neutral - neither positive or negative
- ○ Somewhat negative
- ○ Very negative
- ○ Both positive and negative
- ○ Unsure

Q2.1a Why do you say that?

____

____

____

____

____

Q2.2 **Thinking about your work, how much do the following work issues negatively impact your mental health?**	A great deal	A lot	A moderate amount	A little	Not at all	Not applicable
1/11. Work content/task design - Example: Your work feels meaningless; what you are asked to do is an under use of your skills or you are asked to do tasks above your skill level.	○	○	○	○	○	○
2/11. Workload & work pace - Example: Your workload is too high or too low; you are expected to do your work with challenging deadlines and time pressures; you are compelled to work multiple jobs; staffing levels are unsafe or understaffed.	○	○	○	○	○	○

3/11. Work schedule - Example: Your work schedule is unpredictable, long, and/or inflexible (includes long hours, lack of breaks, bad or unpredictable shift patterns)	○	○	○	○	○	○
4/11. Control - Example: Decision making around your work is made without you, including over your workload and deadlines.	○	○	○	○	○	○
5/11. Environment & equipment - Example: Your work environment and/or equipment is not safe (bad lighting, lack of space, too much noise, lack of rules to keep you safe from mental or physical illness etc.)	○	○	○	○	○	○

6/11. Organizational culture & function - Example: Communication between managers and workers is poor or does not exist.	○	○	○	○	○	○
7/11. Interpersonal relationships at work - Example: Workers are isolated from each other socially or physically; poor relationships with superiors; conflict and/or violence; lack of social support; bullying; harassment	○	○	○	○	○	○
8/11. Role in organization - Example: Role ambiguity; role conflict; lack of clarity around work responsibilities	○	○	○	○	○	○

9/11. Career development - Example: Work insecurity and/or you feel “stuck’ in your work; under promotion or over promotion; poor pay; short or unstable contract agreements; reliance on performance related pay or bonuses	○	○	○	○	○	○
10/11. Work-life balance - Example: Conflicting demands of work and home; low support at home; demands of work make it difficult to spend time with family or friends	○	○	○	○	○	○
11/11. Job stability - Example: Fear of downsizing or being fired or laid off	○	○	○	○	○	○

Q2.2a Are there any other work issues or factors in the workplace that you feel have negatively impacted your mental health and wellbeing?

○ Yes, please specify ____

○ No

**Q2.3 How important is it for mental health supports and programs at work to:**	Extremely important	Very important	Moderately important	Slightly important	Not at all important
1/13. Support workers with mental health issues and challenges such as feelings of anxiety, depression, stress, burnout (including supporting individuals with managing or recovering from mental health issues).	○	○	○	○	○
2/13. Enhance and promote positive mental health including mental well-being, life satisfaction, positive self-concept, and resilience.	○	○	○	○	○
3/13. Reduce work related problems like low work satisfaction, low productivity, high turnover, , and high absenteeism.	○	○	○	○	○
4/13. Identify workers who are experiencing signs and symptoms of self-harm or thoughts of suicide and provide access to programs to help someone in crisis.	○	○	○	○	○
5/13. Recognize signs of the unhealthy use of alcohol or drugs and provide access to programs to help support workers who have problems with substance use.	○	○	○	○	○
6/13. Support a worker’s overall quality of life (helping workers lead healthy and fulfilling lives inside and outside of work).	○	○	○	○	○
7/13. Increase and improve the knowledge, attitudes, and skills **workers** have about mental health to reduce stigma, and so that they can help support their colleagues.	○	○	○	○	○
8/13. Increase and improve the knowledge, attitudes, and skills **managers** have about mental health to reduce stigma, and so that they can help support their colleagues.	○	○	○	○	○
9/13. Increase the ability of workers to seek out mental health programs or support, including at work.	○	○	○	○	○

10/13. Increase the capacity of **<u>managers</u>** to support workers to seek out mental health programs or support, including at work.	○	○	○	○	○
11/13. Work to improve the leadership style of **<u>managers</u>** so that they are better at supporting workers generally.	○	○	○	○	○
12/13. Evaluate **<u>worker</u>** satisfaction with what supports and programs are provided, and how they are provided.	○	○	○	○	○
13/13. Protect workers from being penalized for experiencing or managing mental health issues and/or challenges.	○	○	○	○	○

**Q2.4 How important is it for work to:**	Extremely important	Very important	Moderately important	Slightly important	Not at all important
1/7. Provide access to services and programs to promote good mental health and prevent mental ill-health, including harmful behaviours (e.g., alcohol or drug use, self-harm, suicide)?	○	○	○	○	○
2/7. Train its **managers/supervisors/leaders** to support workers experiencing mental health problems or issues?	○	○	○	○	○
3/7. Protect workers' mental health through changes in a company or organization's policies (e.g., flexible working hours), to minimize risks to mental health at work?	○	○	○	○	○
4/7. Support workers with symptoms of distress / mental health challenges?	○	○	○	○	○
5/7. Support workers on sickness absence for mental health to Return to Work? *(A Return to Work program is a plan that helps injured workers remain at work or safely return to suitable work).*	○	○	○	○	○
6/7. Support the hiring of workers currently and/or with a history of experiencing mental health challenges?	○	○	○	○	○
7/7. To provide access to mental health screening for workers experiencing signs of distress or mental health challenges in order to access care/support?	○	○	○	○	○

Q2.5 What in general are the concerns you have about promoting and supporting mental health, and preventing mental illness for workers?

____

____

____

____

____

Q2.6 What are potential benefits of mental health being promoted and supported, and mental illness prevented, for workers?

____

____

____

____

____

Q2.8 In many places the nature of work is changing. How people work may be changing. When you think about these changes, what issues do you think should be considered for mental health promotion, prevention or support for workers in the future?

____

____

____

____

____

Q2.9 The COVID-19 pandemic has caused changes in work for some people. What issues do you think should be considered for mental health promotion, prevention or support for workers who have experienced changes due to the COVID-19 pandemic?

____

____

____

____

____

Display This Question:

If Q1.15 = Health worker (e.g., physician, nurse, etc.)

Or Q1.15 = Emergency public services work (e.g., firefighting, paramedic, police)

Or Q1.15 = Humanitarian work (national)

Or Q1.15 = Humanitarian work (international)

Q2.10 What are factors uniquely related to your work in health service delivery, emergency response, and/or the humanitarian sector that should be considered when promoting, preventing and supporting work-related mental health?

____

____

____

____

____

Q2.7 What are potential benefits and/or harms around mental health screening at work?

____

____

____

____

____

End of Block: Section 2: Values and Preferences - General Questions

---

Start of Block: Section 3: Intervention Specific - Awareness of Service and Program Types

Q3.1 Section 3 of 4: This section asks for your thoughts on specific types of mental health programs and services at work.

For the next several questions, you will be asked for your thoughts on various types of mental health programs and services at work.

Which of the following mental health services and programs have you heard of before?
(check all that apply)

☐ **1. Psychosocial/emotional** (e.g., talking to a professional, peer support worker or other care provider about feelings of stress, how to manage problems or symptoms of mental illness, or participating in self-help and coaching programs, etc.)

☐ **2. Physical exercise** (e.g., an exercise routine that has been given to you by a doctor or another health professional)

☐ **3. Health promotion** (e.g., combined programmes addressing a worker's lifestyle such as nutrition, physical activity, health education, stress management)

☐ **4. Training/Education** to learn more about mental health, lowering stigma, or building skills to support the mental health and well-being of workers (incl. skills development and leadership coaching)

☐ **5. Screening** to support mental health (identifying signs that someone is facing problems so that they can be supported early)

☐ **6. Return to work** (after sickness absence related to mental health). *A Return to Work program is a plan that helps injured workers remain at work or safely return to suitable work.*

☐ **7. Vocational support** to help people experiencing mental health problems to find and keep work

Q3.1a
Have you ever heard of any work policies or programs that aimed to support worker mental health through organizational changes? (e.g., organization policies or programs that lead to changes in the working environment, working conditions, work tasks for the purpose of supporting workers' mental health including workload and pace, work schedule, control over

work, work environment and safety, organizational culture, relationships with managers and co-workers, career development, work-life balance).

- ◯ Yes
- ◯ No

End of Block: Section 3: Intervention Specific - Awareness of Service and Program Types

---

Start of Block: Section 3: Intervention Specific

Q3.2 ${lm://Field/1}

Do you have access to it at or through work? (includes insurance coverage)

- ◯ Yes
- ◯ No
- ◯ Unsure/Don't Know

Q3.3 ${lm://Field/1}

Have you ever used it? (in your current work, and/or work you have done in the past)

- ◯ Yes
- ◯ No
- ◯ Unsure/ Don't know

Display This Question:

If Q3.3 = No

Q3.3a ${lm://Field/1}

Would you ever use it?

- Yes
- No
- Unsure/Don't Know

Display This Question:

If Q3.3 = No

Q3.4a ${lm://Field/1}

How easy would it be for you to access?

- Very easy
- Easy
- Neutral (neither easy, nor difficult)
- Difficult
- Very difficult
- Unsure/ Don't Know

Display This Question:

If Q3.3 = Yes

Q3.4b
${lm://Field/1}

How easy was it for you to access?

- ○ Very easy
- ○ Easy
- ○ Neutral (neither easy, nor difficult)
- ○ Difficult
- ○ Very difficult
- ○ Unsure/ Don't Know

Q3.5
${lm://Field/1}   What do you feel are the benefits of it?
(check all that apply)

- ☐ It is convenient for the worker
- ☐ It is helpful for the worker’s mental health
- ☐ It removes barriers such as stigma
- ☐ It is affordable for the worker

Display This Choice:

If Q1.16 = Someone supervises me, I supervise one or more people

Or Q1.16 = No one supervises me, I supervise one or more people

- ☐ It is affordable for the company
- ☐ It benefits the morale of the whole organization
- ☐ ⊗There are no benefits of it
- ☐ Unsure/ Don't Know
- ☐ Other, please specify ________________

Q3.6
${lm://Field/1}

What do you feel are the concerns with it?
(check all that apply)

- ☐ Lack of privacy and confidentiality
- ☐ Fear of judgement/stigma
- ☐ It is inconvenient
- ☐ It is difficult to access
- ☐ It is expensive for workers

Display This Choice:

If Q1.16 = Someone supervises me, I supervise one or more people

Or Q1.16 = No one supervises me, I supervise one or more people

- ☐ It is expensive for the company
- ☐ ⊗ I have no concerns
- ☐ Unsure/Don't know
- ☐ Other, please specify

____

Q3.7 ${lm://Field/1}

Given the benefits and concerns, would you recommend it be used at work?

- ○ Yes
- ○ No
- ○ Unsure/Don't Know
- ○ Other, please specify ____

Display This Question:

If Q1.16 = Someone supervises me, I supervise one or more people

Or Q1.16 = No one supervises me, I supervise one or more people

Q3.8
${lm://Field/1}

Have you ever provided a referral, or information about it to workers?
(check all that apply)

- ☐ ⊗Yes
- ☐ No
- ☐ It is not available where I work
- ☐ It is not related to my work
- ☐ Unsure/Don't Know
- ☐ Other, please specify

____

Display This Question:

If Q1.16 = Someone supervises me, I supervise one or more people

Or Q1.16 = No one supervises me, I supervise one or more people

Q3.9 ${lm://Field/1}

How confident and informed do you feel about this type of program or service?
(check all that apply)

- ☐ I feel very confident and informed
- ☐ I need information and/or more information
- ☐ I need training and/or more training
- ☐ Unsure/Don't Know
- ☐ Other, please specify ____

End of Block: Section 3: Intervention Specific

Start of Block: Section 3: Intervention Specific - Organizational Changes

Q3.2z

Organizational changes (e.g., organization policies or programs that lead to changes in the working environment, working conditions, work tasks for the purpose of supporting workers' mental health including workload and pace, work schedule, control over work, work environment and safety, organizational culture, relationships with managers and co-workers, career development, work-life balance).

Do you have access to work policies or programs that aim to support worker mental health through organizational changes?

- ○ Yes
- ○ No
- ○ Unsure/ Don't know

Q3.3z
Organizational changes (e.g., organization policies or programs that lead to changes in the working environment, working conditions, work tasks for the purpose of supporting workers' mental health including workload and pace, work schedule, control over work, work environment and safety, organizational culture, relationships with managers and co-workers, career

development, work-life balance).

Have you ever participated in a work policy or program aiming to support worker mental health through organizational changes?

- Yes
- No
- Unsure/ Don't Know

Display This Question:

If Q3.3z = No

Q3.3za Organizational changes (e.g., organization policies or programs that lead to changes in the working environment, working conditions, work tasks for the purpose of supporting workers' mental health including workload and pace, work schedule, control over work, work environment and safety, organizational culture, relationships with managers and co-workers, career development, work-life balance).

Would you ever participate in a work policy or program aiming to support worker mental health through organizational changes?

- Yes
- No
- Unsure/ Don't Know

Display This Question:

If Q3.3z = Yes

Q3.4za
Organizational changes (e.g., organization policies or programs that lead to changes in the working environment, working conditions, work tasks for the purpose of supporting workers' mental health including workload and pace, work schedule, control over work, work environment and safety, organizational culture, relationships with managers and co-workers, career development, work-life balance).

How easy was it to participate in a work policy or program aiming to support worker mental health through organizational changes?

- Extremely easy
- Somewhat easy
- Neither easy nor difficult
- Somewhat difficult
- Extremely difficult

Display This Question:

If Q3.3z = No

Or Q3.3z = Unsure/ Don't Know

Q3.4zb
Organizational changes (e.g., organization policies or programs that lead to changes in the working environment, working conditions, work tasks for the purpose of supporting workers' mental health including workload and pace, work schedule, control over work, work environment and safety, organizational culture, relationships with managers and co-workers, career development, work-life balance).

How easy would it be to participate in a work policy or program seeking to support worker mental health through organizational changes?

- Extremely easy
- Somewhat easy
- Neither easy nor difficult
- Somewhat difficult
- Extremely difficult
- Unsure/ Don't Know

Q3.5z
Organizational changes (e.g., organization policies or programs that lead to changes in the working environment, working conditions, work tasks for the purpose of supporting workers' mental health including workload and pace, work schedule, control over work, work environment and safety, organizational culture, relationships with managers and co-workers, career development, work-life balance).

What do you feel are the benefits of policies or programs seeking to support worker mental health through organizational changes?
(check all that apply)

- ☐ It is convenient for the worker
- ☐ It is helpful for the worker's mental health
- ☐ It removes barriers such as stigma
- ☐ It is affordable for the worker
- ☐ It is affordable for the company
- ☐ It benefits the morale of the whole organization
- ☐ ⊗ There are no benefits of it
- ☐ Unsure/ Don't Know
- ☐ Other, please specify

____

Q3.6z
Organizational changes (e.g., organization policies or programs that lead to changes in the working environment, working conditions, work tasks for the purpose of supporting workers' mental health including workload and pace, work schedule, control over work, work environment and safety, organizational culture, relationships with managers and co-workers, career development, work-life balance).

What do you feel are the concerns with policies or programs seeking to support worker mental

health through organizational changes?
(check all that apply)

- ☐ Lack of privacy
- ☐ Fear of judgement/stigma
- ☐ It is inconvenient
- ☐ It is difficult to access
- ☐ It is expensive for workers
- ☐ It is expensive for the company
- ☐ ⊗ I have no concerns
- ☐ Unsure/ Don't Know
- ☐ Other, please specify

____

Q3.7z
Organizational changes (e.g., organization policies or programs that lead to changes in the working environment, working conditions, work tasks for the purpose of supporting workers' mental health including workload and pace, work schedule, control over work, work environment and safety, organizational culture, relationships with managers and co-workers, career development, work-life balance).

Given the benefits and concerns, would you recommend this approach be used at work?

- ◯ Yes
- ◯ No
- ◯ Unsure/ Don't Know

Display This Question:

If Q1.16 = Someone supervises me, I supervise one or more people

Or Q1.16 = No one supervises me, I supervise one or more people

Q3.8z
Organizational changes (e.g., organization policies or programs that lead to changes in the working environment, working conditions, work tasks for the purpose of supporting workers' mental health including workload and pace, work schedule, control over work, work environment and safety, organizational culture, relationships with managers and co-workers, career development, work-life balance).

Have you ever provided a referral, or information about ways to get involved in work policies or

programs seeking to support worker mental health through organizational changes? *(check all that apply)*

- ☐ ⊗Yes
- ☐ No
- ☐ It is not available where I work
- ☐ It is not related to my work
- ☐ Unsure/ Don't Know
- ☐ Other, please specify ________________

Display This Question:

If Q1.16 = Someone supervises me, I supervise one or more people

Or Q1.16 = No one supervises me, I supervise one or more people

Q3.9z
Organizational changes (e.g., organization policies or programs that lead to changes in the working environment, working conditions, work tasks for the purpose of supporting workers' mental health including workload and pace, work schedule, control over work, work environment and safety, organizational culture, relationships with managers and co-workers, career development, work-life balance).

How confident or informed do you feel about this type of intervention? *(check all that apply)*

- ☐ I feel very confident and informed
- ☐ I need information and/or more information
- ☐ I need training and/or more training
- ☐ Unsure/ Don't Know
- ☐ Other, please specify ____

End of Block: Section 3: Intervention Specific - Organizational Changes

---

Start of Block: Section 3: Intervention Specific - Concluding Questions

Q3.10 Have you ever considered participating in any of the mental health services or programs that have been mentioned, but did not do so?

- ○ Yes
- ○ No

Display This Question:

If Q3.10 = Yes

Q3.10a Which mental health service or program?
(check all that apply)

◯ **1. Psychosocial/emotional** *(e.g., talking to a professional, peer support worker or other care provider about feelings of stress, how to manage problems or symptoms of mental illness, or participating in self-help and coaching programs, etc.)*

◯ **2. Physical exercise** *(e.g., an exercise routine that has been given to you by a doctor or another health professional)*

◯ **3. Health promotion** *(e.g., combined programs addressing a worker's lifestyle such as nutrition, physical activity, health education, stress management)*

◯ **4. Training/Education** to learn more about mental health, lowering stigma, or building skills to support the mental health and well-being of workers *(incl. skills development and leadership coaching)*

◯ **5. Screening** to support mental health *(identifying signs that someone is facing problems so that they can be supported early)*

◯ **6. Return to work** *(after sickness absence related to mental health). A Return to Work program is a plan that helps injured workers remain at work or safely return to suitable work.*

◯ **7. Skills development** to help people experiencing mental health problems to find and keep work.

◯ **8. Organizational changes** *(e.g., organization policies or programs that lead to changes in the working environment, working conditions, work tasks for the purpose of supporting workers' mental health including workload and pace, work schedule, control over work, work environment and safety, organisational culture, relationships with managers and co-workers, career development, work-life balance).*

Display This Question:

If Q3.10 = Yes

Q3.10b Why did you not participate (check all that apply)?

- ☐ Did not have time (for example due to workload, lack of flexibility of service delivery or time needed to participate)
- ☐ Was not able to access the services or program
- ☐ Was worried about privacy (for example there was no private space to access the service/program or lack of confidential means to address issues)
- ☐ Preferred to manage by myself
- ☐ Did not think it would help/be effective
- ☐ Afraid to ask for help from my manager or employer
- ☐ Afraid my co-workers would find out
- ☐ I was worried that asking for help would lead to negative consequences
- ☐ Did not feel I needed to participate
- ☐ Could not afford it
- ☐ Lack of choice in service types
- ☐ Work is not responsible for supporting or promoting the mental health and wellbeing or workers
- ☐ I am too focused on meeting my basic needs (food, clothing, shelter)
- ☐ Other, please specify ____

End of Block: Section 3: Intervention Specific - Concluding Questions

Start of Block: Section 4: Preferences on Accessibility

JS

. Section 4 of 4: This section asks you about how comfortable you are talking about mental health issues generally, how easy it is for you to access mental health programs and how you would like to receive mental health programs and services if you needed them.

4.1 How comfortable are you to talk about your mental health with the following?

	Very comfortable	Comfortable	Neither comfortable nor uncomfortable	Uncomfortable	Very uncomfortable	Not applicable
1/6. Colleagues	○	○	○	○	○	○
2/6. Managers/supervisors	○	○	○	○	○	○
3/6. Human resources	○	○	○	○	○	○
4/6. Union representatives	○	○	○	○	○	○
5/6. Health services at work (example: in-house occupational health service workers)	○	○	○	○	○	○
6/6. Health services outside of work (example: doctor, psychologist,)	○	○	○	○	○	○

Q4.1a Who would you want support from when…
(check all that apply, then click > next to box for rest of questions)

	Colleagues	Managers/Supervisors	Human Resources	Union Representatives	Health services at work (example: in-house occupational health service workers)	Health services outside of work (example: doctor, psychologist, therapist)
1/4. First experiencing mental health concerns in the workplace?	○	○	○	○	○	○
2/4. Seeking ongoing support for workplace mental health?	○	○	○	○	○	○
3/4. Deciding whether or not to take a leave of absence due to a mental health issue?	○	○	○	○	○	○
4/4. Returning to work after a leave for mental health reasons?	○	○	○	○	○	○

Q4.2 How important is it to access workplace mental health interventions directly on your own (i.e., without the assistance of a manager)?

- ○ Very important (I do not want my employer to know I am accessing services)
- ○ Somewhat important
- ○ Neutral (neither important nor unimportant)
- ○ Somewhat unimportant
- ○ Very unimportant (I do not mind if my employer knows that I am accessing services)
- ○ Unsure
- ○ Depends on the intervention

Q4.3 If you needed it, how would you prefer to access mental health services and programs at work?
(check all that apply)

- ☐ In a group, in person
- ☐ In a group, digitally (online)
- ☐ On my own, in person
- ☐ On my own, digitally (online/self-directed)
- ☐ Other, please specify

____

Q4.4 If you needed it, where would you prefer to receive support?
(check all that apply)

- ☐ At work
- ☐ At home
- ☐ Online
- ☐ Outside of work (other than at home) - Example: in person with a psychologist
- ☐ ⊗I would not be interested in support
- ☐ Other, please specify ________________

Q4.5 If you needed it, from whom would you prefer to receive support?
(check all that apply)

- ☐ A supervisor/superior
- ☐ A human resource or occupational health manager
- ☐ A doctor or other health professional
- ☐ An online app or web-based program
- ☐ No one/ on your own
- ☐ ⊗I would not be interested in support
- ☐ Other, please specify ________________

Q4.6 **What is most important for employers to consider in offering a mental health service or program that is available to workers outside of work?** (drag and drop to rank. (1=most important; 9=least important)
______ Provide time to ensure employees can access the service or program
______ Ensure there is enough physical space between the location of work and the program or service
______ Ensure anonymity in accessing services and programs
______ Provide engaging and interesting content for programs and services
______ Easy to access and use (e.g., convenient, flexible timing)
______ Provide face-to-face intervention options
______ Accessible in multiple ways (e.g., in person, on a computer, on a smartphone)
______ Costs to employee
______ Costs to the employer
______ Evidence based and effective
______ Ensure no resulting negative consequences
______ Other, please specify

End of Block: Section 4: Preferences on Accessibility

Start of Block: Section 5: Additional questions for MANAGERS

Q5.1 Have you ever provided a referral, or information on how or where to access support, to workers/employees for their mental health?

○ Yes

○ No

Display This Question:

If Q5.1 = No

Q5.1a If no, why do you say that? (choose all that apply)

- ☐ It is not available where I work
- ☐ I don't know what is available at my work
- ☐ It is not related to my job
- ☐ Other, please specify ____

Q5.2 How confident do you feel to support workers in distress/experiencing mental health issues or challenges?

- ○ Very confident
- ○ Somewhat confident
- ○ Neutral (neither confident nor unconfident)
- ○ Somewhat unconfident
- ○ Very unconfident

Display This Question:

If Q5.2 = Somewhat confident

Or Q5.2 = Neutral (neither confident nor unconfident)

Or Q5.2 = Somewhat unconfident

Or Q5.2 = Very unconfident

Q5.2a What would help you feel more confident about supporting workers in distress/experiencing mental health issues or challenges? (choose all that apply)

- ☐ I need information or more information about mental health issues
- ☐ I need information or more information about mental health interventions
- ☐ I need training or more training about how to identify and support workers struggling with their mental health
- ☐ I need organizational infrastructure to manage this
- ☐ I feel confident and informed
- ☐ Other, please specify ____

Q5.3 How easily are you able to access TRAINING to improve knowledge, attitudes, skills/behavior to support or improve mental health of workers when needed?
Training: to improve knowledge, attitudes, skills/behavior to support mental health and well-being of workers and/or improve workers' positive mental health and reduce symptoms of mental health conditions, suicide, or substance use.

- ○ I can access when needed
- ○ I am not able to access programs and services
- ○ Other, please specify ____

Q5.4 How would you like to access training?
(check all that apply)

- ☐ In a group, in person
- ☐ In a group, digitally (online)
- ☐ On my own, in person
- ☐ On my own, digitally (online/self-directed)
- ☐ Other, please specify ____

Q5.5 Employee assistance programs are short term counselling services for employees managing challenges at work and home. Do you have training and/or experience in developing and managing employee assistance programs for your employees?

- ◯ Yes
- ◯ No
- ◯ Other, please specify ____

End of Block: Section 5: Additional questions for MANAGERS

Start of Block: Section 6: Additional questions for providers of mental health care for workers

Q6.1 What specific training, information, or skills would you like regarding mental health programs and services at work?
(check all that apply)

- ▢ More information on the programs and services
- ▢ Instructions on next steps to strengthen mental health care/protections for my staff
- ▢ Specific training on mental health services and programs of interest
- ▢ Crisis management
- ▢ Health and safety (e.g., assessing and managing risk at work, putting in prevention measures, developing plans to address it any issues)
- ▢ Evidence about how effective the programs and services are
- ▢ Unsure
- ▢ Other, please specify ____

End of Block: Section 6: Additional questions for providers of mental health care for workers

---

Start of Block: Submit Survey

QEND
End of survey

Would you like to submit your responses? *(If you would like to go back to any questions, you can click the back arrow button below, or you can use the table of contents on the left hand side of the screen (symbol is three horizontal lines)).*

- ○ Yes - submit survey

End of Block: Submit Survey
